# Anthocyanin Boosts Electroactive Biofilms Formation and Regulates Intrinsic Catalytic Activity of Single Cells in *Escherichia coli* for Sustainable Bio-Electrocatalysis in Microbial Fuel Cells

**DOI:** 10.3390/microorganisms14040872

**Published:** 2026-04-13

**Authors:** Kai Zong, Liwen Chen, Waseem Raza, Xin Wang, Lin Yang, Zhongwei Chen

**Affiliations:** 1 Institute of Carbon Neutrality, Zhejiang Wanli University, Ningbo 315100, China; chenliwen0903@163.com (L.C.); razawaseem2@zwu.edu.cn (W.R.); wangx@zwu.edu.cn (X.W.); yanglin@htu.edu.cn (L.Y.); 2 Ningbo Key Laboratory of High Energy Density Battery, Yuyao Innovation Institute, Zhejiang Wanli University, Ningbo 315400, China; 3 Power Battery and System Research Center, Dalian Institute of Chemical Physics, Chinese Academy of Sciences, Dalian 116023, China; 4 State Key Laboratory of Catalysis, Dalian Institute of Chemical Physics, Chinese Academy of Sciences, Dalian 116023, China

**Keywords:** microbial fuel cell, *Escherichia coli*, oxygen reduction reaction, anthocyanin, biofilms, extracellular electron transfer, bioenergy, biocatalyst

## Abstract

Microbial fuel cells (MFCs) are a sustainable approach to wastewater treatment and energy recovery. However, their practical utility is often limited by sluggish cathode kinetics. For this technology, developing cost-effective biocatalysts that do not compromise effectiveness is a primary challenge. In this study, we utilized anthocyanin molecularly functionalized *Escherichia coli* (Cya-WT) to promote the formation of electroactive biofilms and regulate the intrinsic catalytic activity of single cells, thereby enhancing extracellular electron transfer. MFCs incorporating Cya-WT-loaded carbon cloth (CC) biocathodes were configured to simultaneously evaluate power generation and glucose degradation activity. The results indicated that Cya-WT exhibited significantly improved oxygen reduction reaction (ORR) activity, achieving a reduction peak current of 3.61 mA cm^−2^, compared to 2.02 mA cm^−2^ for wild-type *E*. *coli* (WT). The assembled MFC offers a peak power density of 268 ± 13.4 μW cm^−2^ and decomposes 17.1 ± 1.15 mM of glucose in 150 h, maintaining a consistent voltage output for 800 h. These results demonstrate that anthocyanin functionalization significantly enhances the electrocatalytic performance and metabolic capabilities of *E. coli*. This novel catalyst design method offers a new strategy for low-cost, renewable MFC cathode catalysts and shows good promise in MFC biopower generation through the assembly of carbon-based biocathodes.

## 1. Introduction

The continuous and large-scale discharge of wastewater from agricultural, urban, and industrial sources poses a significant threat to aquatic ecosystems and public health [1,2,3]. Since sewage is rich in carbohydrates, such as sugars, bio-electrochemical systems utilizing microorganisms that can metabolize these sugars offer an eco-friendly and sustainable strategy for breaking down organic pollutants [4,5,6]. In this context, novel and sustainable techniques for treating wastewater aim to transform contaminants into valuable resources [7,8].

Microbial fuel cells (MFCs), as bio-electrochemical systems, serve dual functions by simultaneously generating electricity and degrading pollutants, demonstrating strong potential for energy and environmental management [9,10,11,12]. These systems primarily utilize microorganisms as electrocatalysts to convert chemical energy from organic pollutants into electrical energy. These electrocatalysts facilitate complex redox reactions in microbial metabolism, enabling the production of renewable energy [13,14,15]. However, the slow kinetics of cathodic reactions pose a significant challenge to the advancement of MFC technology [16]. Recently, noble metal catalysts have shown notable catalytic efficiency for the oxygen reduction reaction (ORR) in the MFC cathode [17]. Nevertheless, they face limitations regarding sustainability and cost-effectiveness [18]. In contrast, microbial catalysts offer considerable advantages over platinum-based catalysts and enzymes, including lower costs, enhanced stability, and the dual capacity for power generation and pollutant degradation [19]. Furthermore, the self-replicating and self-healing capabilities of microbial cells also facilitate the long-term stability of MFC systems [20].

*Escherichia coli* contains abundant intermembrane cytochrome c (Cyt c) proteins. Their electron transfer capability and hemoglobin-like structure indicate a potential role in unconventional ORR catalysis. The precursor protein of Cyt c was overexpressed via genetic engineering, and then covalently assembled and functionalized with the iron–heme center to produce the Cyt c protein. Subsequently, poly-pyrrole nanomaterials were synthesized in situ on the surfaces of *E. coli* cells to enhance interfacial electron transfer between the bacteria and the electrode [21]. To overcome the low activity of *E. coli*’s native Cyt c protein, Niu et al. modulated the protein structure using iron–tannic acid nanocomplexes. The electrode functioned as an electron donor to facilitate the reduction reaction, thereby enhancing overall electrocatalytic efficiency [22]. The utilization of small molecules to modulate the catalytic activity of the *E. coli* cathode in MFCs has not been documented previously. Previous studies have shown that plant-derived polyphenol-rich products can serve as environmentally friendly electron mediators, aiding in bioenergy extraction. Specifically, aromatic compounds bearing electron-shuttling functional groups, such as ortho- or para-dihydroxyl substituents on a benzene ring, can display stable and reversible electron transfer properties [23,24,25].

Herein, we present a sustainable biocatalyst for MFCs utilizing modulated *E. coli* BL21 (DE3) cells to effectively improve the electrochemical performance. Anthocyanin-functionalized *E*. *coli* (Cya-WT) was employed to promote the formation of electroactive biofilms and regulate the intrinsic catalytic activity of single cells, thereby enhancing extracellular electron transfer. The bioelectricity of MFCs based on a carbon cloth (CC) cathode modified with Cya-WT was compared with a carbon-cloth cathode modified with wild-type *E. coli* (WT, as a control). This study not only suggests a novel approach for sustainable microbial catalyst design but also presents a viable technology for bioenergy.

## 2. Materials and Methods

### 2.1. Materials

Tryptone and yeast extract were purchased from OXOID, Thermo Fisher Scientific (Waltham, MA, USA). Acetone, ethanol absolute, sulfuric acid, and nitric acid were purchased from Sinopharm Chemical Reagent (Shanghai, China). NaCl, Na_2_HPO_4_, KH_2_PO_4_, NH_4_Cl, MgSO_4_, CaCl_2_, D-(+)-Glucose, and Nafion™ perfluorinated resin solution were purchased from Aladdin Scientific (Shanghai, China). Anthocyanin was purchased from Macklin Biochemical Technology Co., Ltd. (Shanghai, China). Glutaraldehyde, 2.5% (EM-grade); PBS, 1× (pH7.2–7.4, 0.01 M, cell Culture); and propidium iodide (PI) were purchased from Solarbio Science & Technology Co., Ltd. (Beijing, China). SYTO™ 9 Green Fluorescent Nucleic Acid Stains was purchased from Invitrogen™, Thermo Fisher Scientific.

### 2.2. Bacterial Culture

The preserved *E. coli* BL21 (DE3) strains were inoculated into 5 mL of Luria–Bertani (LB) liquid medium. The culture was then incubated in a shaker at 37 °C and 180 rpm for at least 12 h. Subsequently, the culture corresponding to an inoculation volume of 1% (*v*/*v*) was transferred into 100 mL of LB medium for further growth. When the final absorbance of the bacterial cultures at 600 nm (OD_600_) is about 1.5, the cells were harvested by centrifugation (4 °C, 6000 rpm, 5 min) for subsequent use.

### 2.3. Preparation of Anthocyanin Molecular-Functionalized E. coli

**Figure 1:**
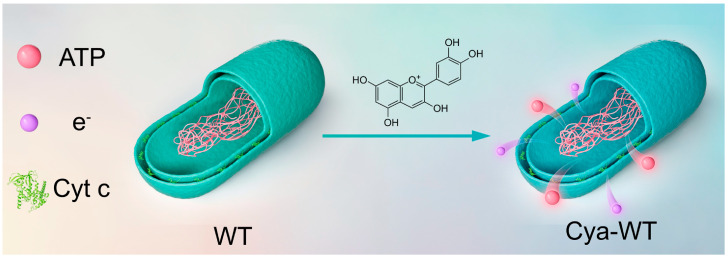
Schematic diagram of Cya-WT synthesis.

The harvested bacterial precipitate was washed twice with ultrapure water, followed by resuspending the bacterial cells in 10 mL of anthocyanin aqueous solution (2 mg mL^−1^). The mixed bacteria solution was strongly swirled at room temperature for 1 min. Proceed by placing the bacterial solution in a shaker at 37 °C and incubating it at 180 rpm for 2 h, the anthocyanin-functionalized *E. coli* (Cya-WT) were obtained by centrifugation (Figure 1).

### 2.4. Scanning Electron Microscope Test

Scanning electron microscopy (SEM, Hitachi, Tokyo, Japan, SU8100) was employed at an accelerating voltage of 3 kV, enabling clear observation of the bacterial sample phenotypes at high magnification. The preparation process for the imaging of bacterial samples in this study involved several steps: bacterial precipitates collected via centrifugation were washed twice with ultrapure water, and then resuspended in 2.5% glutaraldehyde. The samples were fixed at room temperature in the dark for 2 h before being transferred to 4 °C for overnight fixation. Subsequently, the immobilized bacterial samples were eluted using a gradient of 30% to 100% ethanol, dried, and then examined to obtain images.

### 2.5. Transmission Electron Microscope Test

Transmission electron microscopy (TEM, Hitachi, HT7800) was conducted at an accelerated voltage of 80 kV. The high-voltage electron beam penetrates the sample, producing images of varying brightness and darkness, which facilitates high-resolution observation of the sample’s fine morphological features. Given the limited penetrating power of the electron beam, ultrathin sectioning is required for relatively thick samples to accurately observe their internal structures. The sample preparation for TEM followed the same protocol as that for SEM tests.

### 2.6. Confocal Laser Scanning Microscope Test

The SYTO 9/PI double staining technique was primarily employed to assess bacterial viability. 1 mL of bacteria solution cultured to an advanced stage of logarithmic growth was centrifuged (6000 rpm, 5 min). The prepared dyeing solution (500 µL, the SYTO 9 dye, at a working concentration of 5 μM, while the PI dye, at a working concentration of 2 μg mL^−1^) was added to the bacterial suspension and incubated at room temperature for 15 min away from light. The stained cells were detected by a confocal laser scanning microscope (CLSM, Zeiss, Oberkochen, Germany, LSM 900).

### 2.7. Oxygen Adsorption Capacity Test

O_2_ was flowed into a sealed five-port electrolytic cell containing 150 mL of M9 medium (pH 7) composed of 1.02 g Na_2_HPO_4_, 0.45 g KH_2_PO_4_, 0.075 g NaCl, 0.15 g NH_4_Cl, 0.036 g MgSO_4_, and 0.0015 g CaCl_2_ until the oxygen concentration reached 19 mg L^−1^. Then, 1 mL of bacterial liquid was added to the electrolyte to monitor the change in oxygen concentration over time by optical dissolved oxygen sensors (Vernier, Beaverton, OR, USA, LabQuest3).

### 2.8. Pretreatment of Carbon Cloth

The cell pellet obtained by centrifugation of 50 mL bacterial solution was resuspended in 50 mL fresh LB medium. and then several pieces of CC purified with acetone, anhydrous ethanol, concentrated H_2_SO_4_, concentrated HNO_3_, and ultrapure water were immersed in the cell suspension. Afterwards, the mixture was placed in a shaker at 37 °C and 180 rpm for 27 h to an ultimate bacterial solution’s OD_600_ of about 2.2. So, the *E. coli* cells were firmly attached to the carbon cloth in the way of natural growth attached on the CC in the way of natural growth pattern.

### 2.9. Electrochemical Analysis

The electrochemical performance measurements were conducted on an electrochemical workstation (CH Instruments Inc., Austin, TX, USA, CHI760E). Nitrogen (N_2_) was flowed into a sealed five-port electrolytic cell containing 150 mL of M9 for 30 min to achieve saturation. The system was maintained at 37 °C using a water bath. Cyclic voltammetry (CV) was performed under N_2_-saturated conditions after the system stabilized. In this three-electrode configuration, a platinum sheet was taken as the counter electrode, and the reference electrode was a saturated silver chloride electrode. Meanwhile, a CC electrode loaded with bacteria was taken as the working electrode with a scanning rate of 50 mV s^−1^. Oxygen (O_2_) was introduced for 30 min to reach saturation to further perform the CV test under O_2_-saturated conditions at scanning rates of 10, 20, 30, 40, and 50 mV s^−1^. Linear scanning voltammetry (LSV) tests were performed at a scanning rate of 5 mV s^−1^ for electrochemical stability. Both CV and LSV tests were conducted over a voltage range of −0.6 to 0.3 V. Furthermore, electrochemical impedance spectroscopy (EIS) tests were performed under open-circuit conditions. The duration of the current-time (I-T) test is set at 300,000 s.

### 2.10. Microbial Fuel Cell Device Setup

The performance of the MFC was evaluated using a classic H-type dual-chamber reactor, which has an internal working volume of 50 mL and is separated by a proton exchange membrane (PEM). CC with an electroactive area of 4 cm^2^ served as the substrate for the cathode and anode catalysts. The electrolyte in the cathode chamber consisted of M9 medium supplemented with 4 g L^−1^ glucose. O_2_ was introduced for 30 min until saturation was achieved. The electrolyte in the anode chamber comprised M9 medium containing 1 M glucose, with a flow tube connected to pass N_2_ for 30 min until saturation. The cathode catalyst utilized an *E. coli* microbial electrocatalyst affixed to a CC electrode, while the anode catalyst was a commercial 40% Pt/C catalyst with a loading of 2 mg cm^−2^. The open-circuit voltage of the MFC was measured using an electrochemical workstation. The stable open-circuit voltage under varying load resistances was recorded by adjusting the external resistor from 10 to 1 MΩ. The polarization and power density curves of the MFC were plotted with current density on the *x*-axis and voltage and power density values on the *y*-axis. The stable output voltage of the MFC during prolonged operation was collected using a Keithley digital multimeter.

### 2.11. Glucose Degradation Capacity Test

Under the operational conditions of the MFC, the glucose degradation capacity of various *E. coli* strains was assessed. A glucose content detection kit (Solarbio, Beijing, China, BC5880) was employed to measure the glucose concentration at the MFC cathode over time. An ultraviolet–visible spectrophotometer (Shimadzu, Kyoto, Japan, UV-2600i) was used to quantify the characteristic absorption peak at 505 nm. Referencing the standard curve derived from the absorption values of the standard sample under these conditions, the glucose concentration at the MFC anode at various time points was further calculated.

### 2.12. Determination of Adenosine Triphosphate Content

An ATP content determination kit (Mlbio, Vancouver, WA, USA, ml092826) was employed to quantify the ATP levels in different *E. coli* strains. Creatine kinase catalyzes the reaction between creatine and ATP, resulting in the formation of creatine phosphate. The concentration of creatine phosphate was quantified using the phosphomolybdic acid colorimetric method at 700 nm, thereby reflecting the ATP content.

### 2.13. Determination of Protein Content in Biological Membranes

The BCA protein concentration assay kit (Solarbio, PC0020) was employed to quantify the protein content in biofilms formed by various *E. coli* strains grown on CC. In alkaline conditions, the protein reduces Cu^2+^ to Cu^+^, resulting in the formation of a purple-blue complex with the BCA reagent. The absorbance at 562 nm is measured and compared to a standard curve. Using the standard curve derived from the absorption values of the standard substance under these conditions, we can calculate the concentration of the protein being analyzed. The CV curves of *E. coli* before and after the functionalization with anthocyanin molecules were normalized by utilizing the calculated content of biofilms proteins. The current density (mA cm^−1^) was divided by the protein mass (mg cm^−1^) to derive the electrogenic activity per unit biomass (mA mg^−1^, protein).

## 3. Results and Discussion

### 3.1. Analysis of Biological Characteristics

**Figure 2:**
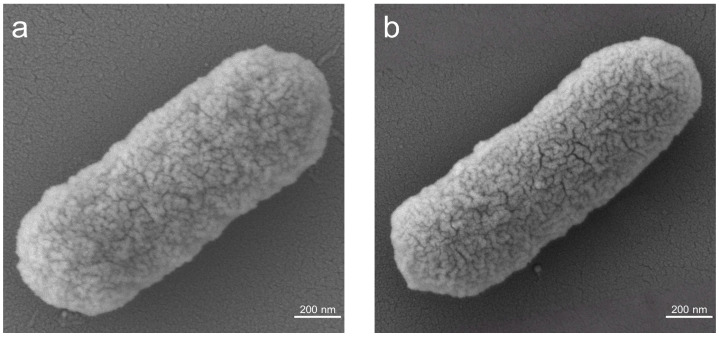
SEM images of (**a**) wild-type *E*. *coli* (WT) and (**b**) Cya-WT.

To investigate the impact of anthocyanin molecular functionalization on the phenotype of *E*. *coli*, the morphology of *E. coli* cells before and after the functionalization process was characterized. SEM images (Figure 2a,b) reveal that both pre- and post-functionalization *E. coli* cells exhibit plump oval characteristics, suggesting that the functionalization step does not significantly alter the phenotype of the cells.

**Figure 3:**
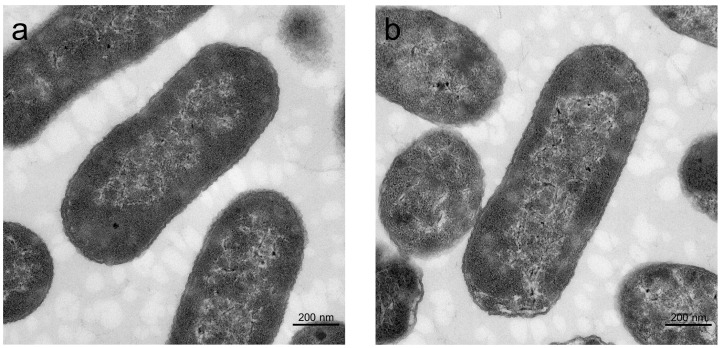
TEM images of (**a**) WT and (**b**) Cya-WT.

TEM images (Figure 3a,b) indicate no apparent changes in the cell membrane or internal structure of *E. coli* following anthocyanin molecular functionalization. These findings demonstrate that the functionalization of anthocyanin molecules does not affect the external morphology or internal structure of *E. coli* cells, thereby supporting their biological activity.

**Figure 4:**
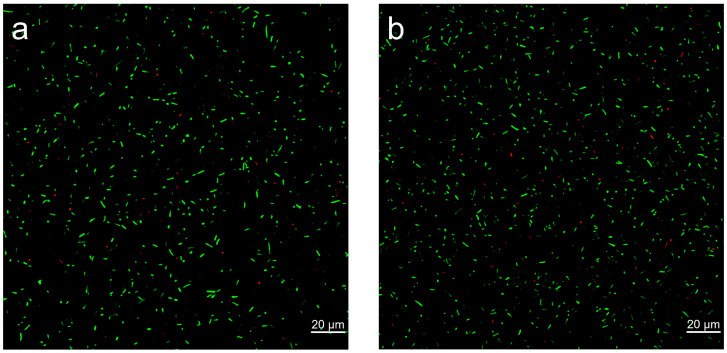
CLSM images of (**a**) WT and (**b**) Cya-WT.

CLSM images (Figure 4a,b) further reveal that both WT and Cya-WT display strong green fluorescence alongside weak red fluorescence. Following treatment with Imaris (Figure S1), the survival rates for WT and Cya-WT were recorded as 98.9% and 99.3%, respectively. These findings suggest that the functionalization of anthocyanin molecules has a negligible impact on the viability of *E. coli* cells.

**Figure 5:**
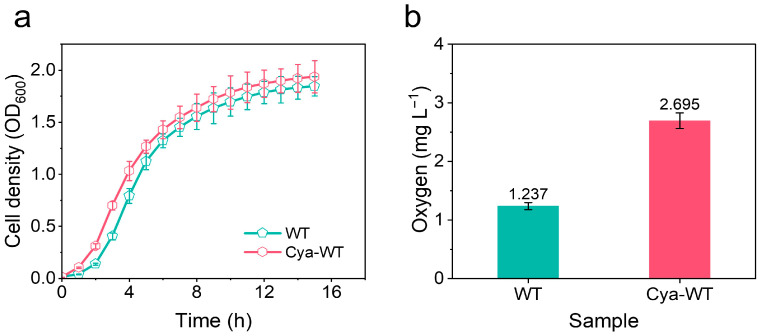
(**a**) Growth curve and (**b**) oxygen balance histogram of WT and Cya-WT.

The growth and reproductive capabilities of various *E. coli* strains were assessed by measuring OD_600_ over time and plotting the growth curves, replicating 3 times (*n* = 3, Figure 5a). Both Cya-WT and WT exhibited good division, and reproduction was observed. Furthermore, a comparison of the OD_600_ values at the same time point revealed that the OD_600_ value for Cya-WT exceeded that of WT, indicating that Cya-WT had a higher cell concentration and enhanced proliferation capacity at that time.

O_2_ acts as the substrate for microbial electrocatalytic ORR. The adsorption capacity of *E. coli* cells for O_2_ significantly influences their electrocatalytic ORR activity. Consequently, the O_2_ consumption capacity of *E. coli* before and after the functionalization with anthocyanin molecules was examined. As illustrated in Figure 5b (*n* = 3), Cya-WT (2.695 ± 0.1348 mg L^−1^) demonstrates a faster O_2_ consumption rate compared to WT (1.237 ± 0.06185 mg L^−1^), suggesting that Cya-WT possesses a greater O_2_ absorption capacity. This enhancement can increase the O_2_ concentration within the cells, thereby facilitating the rapid progression of the ORR process.

### 3.2. Electrochemical Performance of a Half-Cell

To demonstrate the functionality of *E. coli* cells before and after the functionalization with anthocyanin molecules, a series of electrochemical performance tests were conducted. Initially, CV tests were performed in a N_2_-saturated atmosphere To analyze the surface electrochemical characteristics of the microbial catalyst. As illustrated in Figure S2, both *E. coli* samples, prior to and following anthocyanin functionalization, exhibit distinct redox peak signals when compared to the CC electrode. This finding indicates that *E. coli*, as a microbial electrocatalyst, is capable of undergoing a direct electron transfer process with the electrode surface.

**Figure 6:**
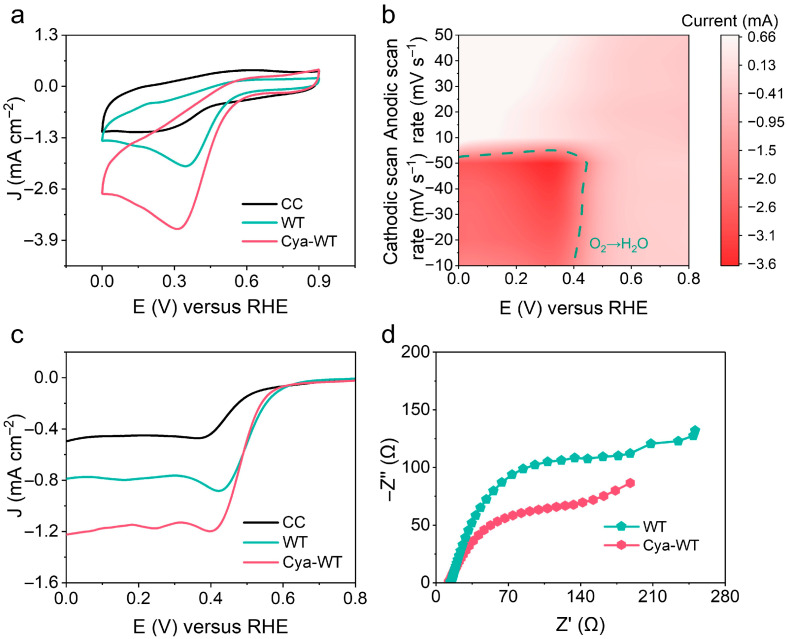
(**a**) CV patterns of the CC electrode, WT cell, and Cya-WT cell in O_2_-saturated solution. (**b**) CV pattern of Cya-WT cell at 10, 20, 30, 40, and 50 mV s^−1^ in O_2_-saturated solution. (**c**) LSV curves of the CC electrode and different *E. coli* cells. (**d**) EIS plots of different *E. coli* cells.

Furthermore, the electrocatalytic performance of various *E. coli* strains was assessed and optimized in O_2_-saturated solutions for ORR. The pronounced reduction peak signal depicted in Figure 6a demonstrates that O_2_ can be electrochemically reduced by *E. coli* both before and after functionalization with anthocyanin molecules. Notably, the reduction peak current for Cya-WT increased from 2.02 mA cm^−2^ for WT to 3.61 mA cm^−2^, reflecting an approximate enhancement of 78.7%. This finding indicates that anthocyanin molecules significantly enhance the electrocatalytic ORR activity of *E. coli* cells.

Secondly, CV tests on *E. coli* both before and after the functionalization with anthocyanin molecules at various scanning rates were performed (Figure 6b). Linear fitting of the scanning rate against the peak current was conducted. As depicted in Figure S3, the peak current exhibits a strong linear correlation with the scanning rates, as evidenced by a correlation coefficient of R^2^ = 0.9981, indicating the surface-controlled electrochemical characteristics of the ORR process.

As shown in Figure 6c, LSV measurements demonstrated a substantial enhancement in the electrocatalytic activity of Cya-WT, with a limiting current density of 1.22 mA cm^−2^, markedly exceeding that of WT, which exhibited a limiting current density of 0.787 mA cm^−2^.

EIS was performed to investigate the interfacial electron transfer properties between various *E. coli* cells and electrodes (Figure 6d). In comparison to WT, Cya-WT exhibits a reduced charge transfer resistance, suggesting that anthocyanin molecules facilitate electron transfer between cells and electrodes, thereby promoting the progression of electrocatalytic reactions.

### 3.3. Power Generation and Glucose Degradation

**Figure 7:**
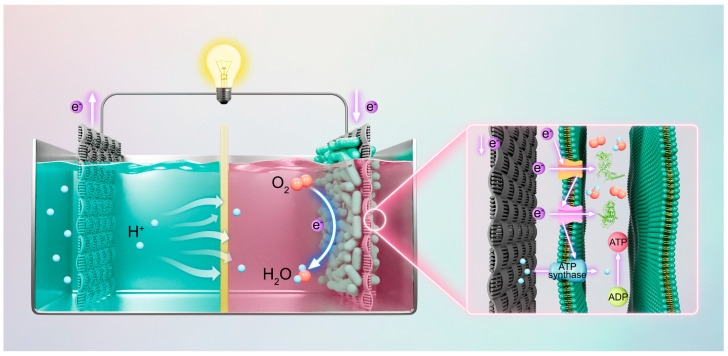
Schematic diagram of the dual-chamber MFC device and its associated catalytic activity mechanism.

To assess the power generation capacity of MFCs constructed with CC biocathodes loaded with various strains of *E. coli*, CC as the substrate, different *E. coli* cells as cathodic biocatalysts, and 40% Pt/C catalysts as anode catalysts (2 mg cm^−2^ for the anode catalysts) were utilized [26]. A dual-chamber MFC device was assembled for this purpose (Figure 7). The operational principle of the MFC involves the Pt/C catalyst facilitating the oxidation of glucose at the anode, which generates electrons. These electrons are subsequently transferred to the cathode via an external circuit, where *E. coli* cells absorb the electrons and catalyze the reduction in O_2_ to produce water (H_2_O) [27]. Electrons are efficiently transferred to the Cyt c protein through the transport pathway of the outer membrane conductive protein. Concurrently, O_2_ molecules penetrate the cell through molecular channel proteins and are reduced to H_2_O under the catalytic influence of Cyt c protein [28]. A 1 M glucose solution was employed as a model pollutant.

As illustrated in Figure S4, the output voltages of various MFCs consistently increase with operating time, ultimately stabilizing at nearly constant values after a certain duration, indicating the successful establishment of functional MFCs. Notably, the startup time for the MFCs utilizing CC biocathodes loaded with Cya-WT is approximately 5.9 h, which is significantly shorter than that for the MFCs employing CC biocathodes loaded with WT, estimated at around 7.0 h. Furthermore, the initial voltage of Cya-WT (~0.429 V) exceeds that of WT (~0.225 V), suggesting that Cya-WT possesses a greater biopower generation capacity.

**Figure 8:**
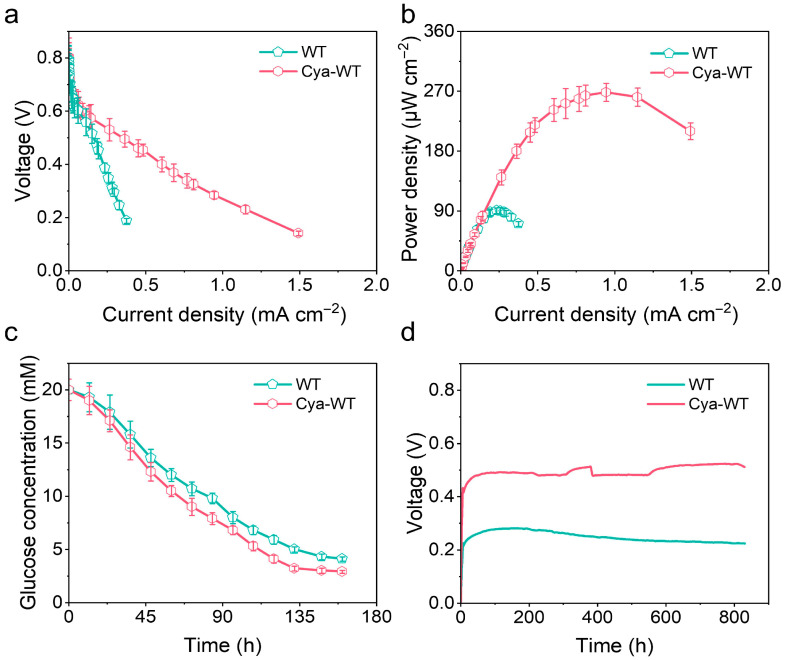
(**a**) Polarization curve diagram, (**b**) power density diagram, and (**c**) glucose degradation test of different *E. coli* in the cathode chamber under MFC operating conditions. (**d**) Long-term stability test plot of MFC assembled using CC biocathodes loaded with different *E. coli* strains.

When the output voltage of the MFCs stabilized, a resistance wire ranging from 10 to 1 MΩ between the anode and cathode was connected. By varying the external resistance, the stable voltage of the MFCs with different external resistors was recorded (*n* = 3). Subsequently, the current was calculated using Ohm’s law, and the polarization curve (Figure 8a) and power density (Figure 8b) of the MFCs were plotted. With this analysis, the power generation capacity of MFCs assembled with CC biocathodes loaded with different strains of *E. coli* can be quantitatively evaluated. According to the polarization curve presented in Figure 8a, the MFCs constructed with CC biocathodes containing various *E. coli* strains exhibit similar open-circuit voltages of 0.8 V. However, the maximum output current of the MFC assembled with CC biocathodes loaded with Cya-WT is 1.49 ± 0.179 mA cm^−2^, which is significantly higher than that of the WT, recorded at 0.375 ± 0.0939 mA cm^−2^. The slope of the polarization curve for Cya-WT is notably smaller than that of WT. The power density curve reveals that the MFC utilizing the CC biocathode of Cya-WT achieves a maximum power density of 268 ± 13.4 μW cm^−2^, surpassing the 90.3 ± 4.52 μW cm^−2^ achieved by the MFC with the CC biocathode of WT. This enhancement is attributed to the functionalization of anthocyanin molecules, which provides *E. coli* cells with highly efficient catalytic activity, along with the conductivity of the cell membrane conferred by Cyt c.

To further assess the extracellular electron transfer efficiency of various *E. coli* strains, EIS tests on MFCs constructed with CC biocathodes containing different *E. coli* strains were performed. The EIS results (Figure S5) indicated that Cya-WT exhibited not only a reduced interfacial transfer resistance but also a lower solution resistance in comparison to the WT. The MFC cathode chamber culture medium, which utilized a CC biocathode loaded with Cya-WT, demonstrated enhanced electron transfer efficiency both among the bacteria and between the bacteria and the electrodes.

Given that the growth of *E. coli* cells necessitates a continuous supply of nutrients, glucose consumption in the cathode chamber during the discharge of MFCs assembled with CC biocathodes containing different *E. coli* strains was concurrently monitored, repeating 3 times. As shown in Figure 8c, Cya-WT demonstrates a higher glucose consumption rate compared to WT. Over the 150 h period, glucose consumption reached 17.1 ± 1.15 mM for Cya-WT, surpassing the 15.9 ± 1.21 mM observed for WT. This finding indicates that Cya-WT possesses a greater metabolic capacity.

To evaluate the long-term operational stability of various *E. coli* cell catalysts, the voltage output performance of MFCs constructed with CC biocathodes containing different *E. coli* strains during extended operation was assessed. As illustrated in Figure 8d, the MFCs utilizing CC biocathodes with three distinct *E. coli* strains exhibited stable voltage output for over 800 h under continuous nutrient supplementation conditions. Furthermore, the output voltage of Cya-WT was measured at 0.497 V, significantly surpassing the 0.239 V observed in the WT group. This finding suggests that the Cya-WT cell catalysts possess considerable potential as a sustainable bioenergy source, and their renewability supports the sustainable development of bio-electrocatalytic energy systems.

### 3.4. Catalytic Activity Mechanism

In nature, most microorganisms create a relatively closed electrochemical environment within their cells due to the insulating properties of their cell wall outer membranes [29]. Electrons generated from substrate oxidation inside the cells are progressively transferred along the electron transport chain (or respiratory chain) to terminal electron acceptors, such as O_2_ or various intermediate metabolites (Figure 7). This process is coupled with protons for transmembrane transport across the cell membrane, resulting in the formation of a transmembrane proton potential that drives the synthesis of ATP. This mechanism fulfills the energy requirements necessary for cellular growth and metabolic processes [30,31]. Research by Xu et al. indicated that anthocyanins, acting as redox mediators, possessed electron-shuttling properties that could possibly facilitate electron transfer on cell membranes, thereby influencing the electron transport chain [32].

**Figure 9:**
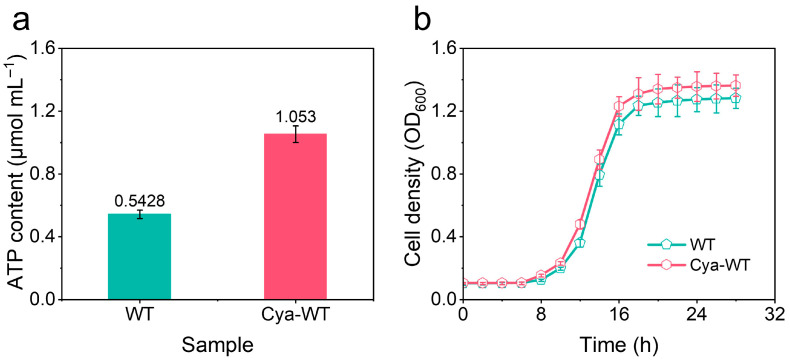
(**a**) Graphs of ATP content determination in different *E. coli* strains. (**b**) Growth curve of different *E. coli* strains under MFC operating conditions.

ATP is abundantly found in *E. coli* cells and functions as their energy currency. It plays a critical role in various metabolic reactions within the organism as a primary energy source [33]. In this study, an ATP content assay kit was employed to quantify the ATP levels in *E. coli* before and after the functionalization with anthocyanin molecules, replicating 3 times as shown in Figure 9a. Understanding the energy metabolism state of *E. coli* cells is essential for analyzing the mechanisms underlying catalytic activity. The data presented in Figure 9a indicate that the ATP content in Cya-WT is 1.050 ± 0.0527 μmol mL^−1^, which exceeds that of WT at 0.543 ± 0.0271 μmol mL^−1^. This result is reproducible across multiple measurements, suggesting that anthocyanin functionalization enhances the metabolic activity of *E. coli* cells.

The growth and reproductive capacity of *E. coli* in the cathode chamber, both before and after the functionalization of anthocyanin molecules under the operational conditions of MFCs constructed with CC biocathodes containing various *E. coli* strains, were assessed. OD_600_ at different time points and plotted the growth curves, which were measured (as illustrated in Figure 9b, *n* = 3). The results revealed that the proliferation rate of Cya-WT exceeded that of WT. This enhancement was attributed to the functionalization of anthocyanin molecules, which improved the electrode respiration of *E. coli* cells, increased metabolic flux, promoted ATP synthesis, and consequently accelerated cell proliferation.

**Figure 10:**
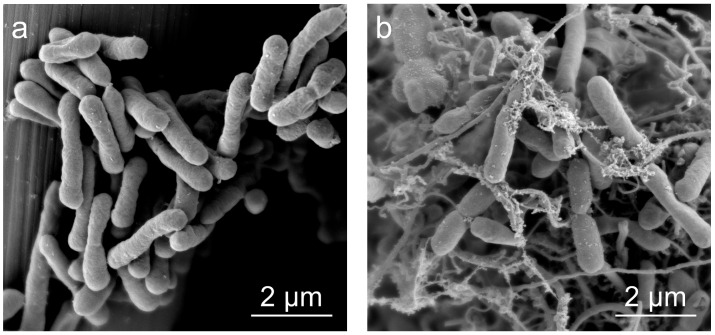
SEM images of biofilms formed by the growth of (**a**) WT and (**b**) Cya-WT on CC.

The growth of *E. coli* cells on CC before and after functionalization was examined using SEM to investigate the impact of anthocyanin molecule functionalization on the growth and reproductive capacity of *E. coli.* The SEM images (Figure 10a,b) reveal that *E. coli* cells, both prior to and following anthocyanin functionalization, growing in situ on the CC, exhibited a robust oval morphology that enhances their biological activity. Additionally, the adhesion of Cya-WT to the CC electrode surpassed that of WT. This observation suggests that anthocyanin functionalization accelerates cell growth, thereby facilitating the colonization of *E. coli* cells on the CC electrode.

**Figure 11:**
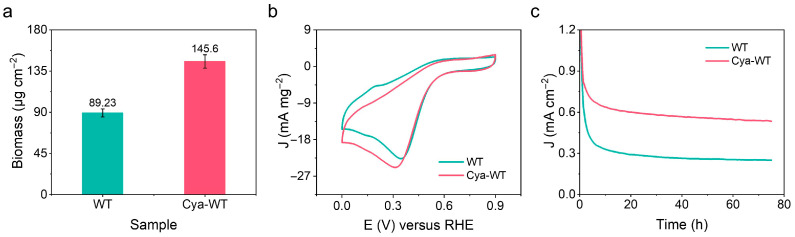
(**a**) Protein content determination diagram for biofilms formed by different *E. coli* strains on carbon fiber cloth. (**b**) CV curve of biomass normalization. (**c**) I-T test diagram of different *E. coli* strains.

Biofilms are intricate structures composed of microorganisms and the EPSs they secrete. Proteins serve as essential components of both microbial cells and EPSs. A BCA protein concentration assay kit was employed to quantify the protein content of communities of bacterial cells formed by the growth of various *E. coli* strains on CC. The concentration of the protein was calculated based on the standard curve derived from the absorbance values of the standard substance under these conditions (Figure 11a, *n* = 3). The protein content of the bacterial communities formed by Cya-WT cells on CC is 145.6 ± 7.28 μg cm^−2^, which exceeds that of WT cells (89.23 ± 4.46 μg cm^−2^). This finding suggests that Cya-WT has a greater biomass on CC.

The CV curves of *E. coli* before and after functionalization with anthocyanin molecules were normalized, utilizing the calculated content of biological membrane proteins. The current density (mA cm^−1^) was divided by the protein mass (mg cm^−1^) to derive the electrogenic activity per unit biomass (mA mg^−1^, protein). This approach allows for a comparison of the electrocatalytic efficiency of each cell under varying conditions. As shown in Figure 11b, the reduction peak current of Cya-WT increased from 22.7 mA mg^−2^ for WT to 24.8 mA mg^−2^, representing an approximate increase of 9.25%. This finding suggests that Cya-WT exhibits higher intrinsic electrocatalytic activity compared to WT cells. Furthermore, it indicates that the electrocatalytic efficiency of MFC catalysts is influenced not only by the colonization density of microbial cells on the electrode but also by the intrinsic electrochemical activity of individual microbial cells. In the three-electrode system assembled with CC biocathodes loaded with different *E. coli* strains, the I-T curves of WT and Cya-WT at a constant potential are presented in Figure 11c. Over a continuous output period of 300,000 s, the current density of Cya-WT significantly increased (~0.6 mA cm^−2^) compared to WT (~0.3 mA cm^−2^), indicating that anthocyanin molecules can be effectively utilized by *E. coli* to enhance its extracellular electron transfer process.

This study focuses on the production of biosafe *E*. *coli* through anthocyanin molecular functionalization using chemical engineering techniques. The research investigates the electrochemical performance and catalytic activity mechanisms of this engineered organism, which serves as a carbon-based biocathode catalyst for MFCs. This whole-cell biohybrid system promotes biofilms formation through naturally occurring bioactive substances, and regulates the metabolic activity of individual cells. It effectively increases current output at the single-cell level, enabling efficient electrocatalysis by *E. coli*. It shows good promise in MFC biopower generation through the assembly of carbon-based biocathodes, achieving an upper-middle level within the industry and providing a simple and practical method (Table 1 and Table 2).

In this study, a preliminary connection between metabolic activity and electrocatalytic performance was established. However, it remains to be investigated whether anthocyanins influence the gene expression involved in cytochrome c synthesis, as well as the interaction between anthocyanin molecules and the active site of the cytochrome c protein. Future studies could utilize techniques such as real-time quantitative polymerase chain reaction (qPCR) to detect the expression levels of key genes associated with electron transport (e.g., Cyt c) and biofilms formation, and to determine whether the system significantly upregulates these genes [44]. Circular dichroism spectroscopy (CDS) could be employed to observe conformational changes in the secondary structure of Cyt c [45]. Additionally, X-ray photoelectron spectroscopy (XPS) and nuclear magnetic resonance spectroscopy (NMRS) can be utilized to examine changes in the coordination structure of the heme active center in Cyt c [46]. Density functional theory (DFT) calculations may serve as a valuable complement to these experimental approaches [47]. Furthermore, the mechanism underlying the enhanced electrocatalytic ORR activity in *E. coli* functionalized with anthocyanin molecules warrants elaboration. Structural biology techniques, such as cryo-electron microscopy (Cryo-EM), can provide insights into the structural and conformational changes in proteins [48].

MFCs can perform dual functions: biopower generation and pollutant degradation, by harnessing the respiration of microbial cells. However, the research presented in this paper remains at the foundational stage, focusing on laboratory studies involving single cells and glucose as model pollutants. To align this research more closely with practical application scenarios, we plan to design a battery device system as an MFC stack for testing in future work, utilizing actual domestic sewage for microbial culture. This approach will advance our project toward the application research stage [49].

## 4. Conclusions

In this study, a novel microbial catalyst, Cya-WT, which serves dual purposes in power generation and pollutant degradation, was proposed. This microbial catalyst performed well in the electrocatalytic process of the ORR and maintained stable voltage output performance for over 800 h. The catalyst is environmentally friendly and renewable, eliminating the need for toxic chemicals and enzymes that are commonly used in industrial processes. Notably, the designed MFC with a CC biocathode loaded with Cya-WT exhibits good bioenergy potential, characterized by its rapid glucose consumption. Anthocyanin molecules promote the formation of electroactive biofilms and regulate the intrinsic catalytic activity of single cells, enabling sustainable bio-electrocatalysis in MFCs. This novel catalyst design method offers a new strategy for low-cost, renewable MFC cathode catalysts.

## Figures and Tables

**Table 1:** Comparison of reported power densities for several reported biofuel cells.

Anode Catalyst	Cathode Catalyst	Power Density (μW cm^−2^)	Reference
Pt/C	Engineered red blood cells	21.11	[34]
Yeast modified with enzymes	Laccase	36.1	[35]
Sludge	White rot fungi	4.13	[36]
Activated sludge	Fe-NC@CBC	64.1	[37]
Glucose oxidase	Hyaluronate-Au@Pt	15.8	[38]
Pt/C	*E. coli* cell@Fe-TA	301	[22]
Pt/C	Genetically engineered *E. coli* cell@PPy	334	[26]
Pt/C	Engineered *Bacillus natto* @sPDA	412	[39]
Pt/C	Cya-WT	268	This work

**Table 2:** Comparison of reported glucose consumption levels for several reported MFCs.

Microorganism	Consumption Level Within a Limited Time (mM)	Reference
*Rhodoferax ferrireducens*	0.3	[40]
Genetically engineered *Shewanella oneidensis*	12.1	[41]
*Saccharomyces cerevisiae -S. oneidensis*	11	[42]
*S. cerevisiae-S. oneidensis*	3.3	[43]
*E. coli* cell@Fe-TA	18	[22]
Genetically engineered *E. coli* cell@PPy	19.4	[26]
Cya-WT	17.1	This work

## Data Availability

The original contributions presented in this study are included in the article/Supplementary Material. Further inquiries can be directed to the corresponding authors.

## Supplementary Materials

The following supporting information can be downloaded at https://www.mdpi.com/article/10.3390/microorganisms14040872/s1. Figure S1. The survival rate graph was obtained based on Imaris processing of WT and Cya-WT; Figure S2. CV patterns of the CC electrode, WT cell, and Cya-WT cell in N_2_-saturated solution; Figure S3. Linear relationship plot between cathode peak current and scanning rate; Figure S4. Startup voltage diagram of the MFC assembled using the CC biocathodes loaded with different *E. coli* loads; Figure S5. EIS diagram of the MFC assembled using the CC biocathodes loaded with different *E. coli* loads.

## Abbreviations

The following abbreviations are used in this manuscript:

MFCs	Microbial fuel cells
ORR	Oxygen reduction reaction
*E**. coli*	*Escherichia coli*
Cyt c	Cytochrome c
CC	Carbon cloth
Cya-WT	Anthocyanin molecular-functionalized *Escherichia coli*
PI	Propidium iodide
CV	Cyclic voltammetry
LSV	Linear scanning voltammetry
EIS	Electrochemical impedance spectroscopy
WT	Wild-type *Escherichia coli*
OD_600_	Absorbance of the bacterial cultures at 600 nm
N_2_	Nitrogen
O_2_	Oxygen
I-T	Current-time
PEM	Proton exchange membrane
SEM	Scanning electron microscopy
TEM	Transmission electron microscopy
CLSM	Confocal laser scanning microscopy
ATP	Adenosine triphosphate
EPS	Extracellular polymers
H_2_O	Water
*Bacillus natto*	*B. natto*
*Rhodoferax ferrireducens*	*R. ferrireducens*
*Shewanella oneidensis*	*S. oneidensis*
qPCR	Real-time quantitative polymerase chain reaction
CDS	Circular dichroism spectroscopy
XPS	X-ray photoelectron spectroscopy
NMRS	Nuclear magnetic resonance spectroscopy
DFT	Density functional theory
Cyro-EM	Cryo-electron microscopy

## References

[1] Schulte-Uebbing L.F., Beusen A.H.W., Bouwman A.F., de Vries W. (2022). From planetary to regional boundaries for agricultural nitrogen pollution. Nature.

[2] Schwabe K., Nemati M., Amin R., Tran Q., Jassby D. (2020). Unintended consequences of water conservation on the use of treated municipal wastewater. Nat. Sustain..

[3] Lu L., Guest J.S., Peters C.A., Zhu X., Rau G.H., Ren Z.J. (2018). Wastewater treatment for carbon capture and utilization. Nat. Sustain..

[4] Wei W., Sun P., Li Z., Song K., Su W., Wang B., Liu Y., Zhao J. (2018). A surface-display biohybrid approach to light-driven hydrogen production in air. Sci. Adv..

[5] Guo J., Suástegui M., Sakimoto K.K., Moody V.M., Xiao G., Nocera D.G., Joshi N.S. (2018). Light-driven fine chemical production in yeast biohybrids. Science.

[6] Chen Y.-d., Wang R., Duan X., Wang S., Ren N.-q., Ho S.-H. (2020). Production, properties, and catalytic applications of sludge derived biochar for environmental remediation. Water Res..

[7] Feng C., Bonetti L., Lu H., Zhou Z., Lotti T., Jia M., Rizzardi G., De Nardo L., Malpei F. (2024). Extracellular polymeric substances as paper coating biomaterials derived from anaerobic granular sludge. Environ. Sci. Ecotechnol..

[8] Cha Q.-Q., Liu S.-S., Dang Y.-R., Ren X.-B., Xu F., Li P.-Y., Chen X.-L., Wang P., Zhang X.-Y., Zhang Y.-Z. (2023). Ecological function and interaction of different bacterial groups during alginate processing in coastal seawater community. Environ. Int..

[9] Liu H., Yao Y., Yuan X., Hui J., An W., Xu S., Zhang Y. (2026). Advances and challenges in synergistic fenton-microbial fuel cell systems for emerging contaminants removal: Mechanisms, configurations, and applications. Bioresour. Technol..

[10] Keasling J., Garcia Martin H., Lee T.S., Mukhopadhyay A., Singer S.W., Sundstrom E. (2021). Microbial production of advanced biofuels. Nat. Rev. Microbiol..

[11] Liu S., Wang A., Liu Y., Zhou W., Wen H., Zhang H., Sun K., Li S., Zhou J., Wang Y. (2024). Catalytically active carbon for oxygen reduction reaction in energy conversion: Recent advances and future perspectives. Adv. Sci..

[12] Li F., Zhang B., Long X., Yu H., Shi S., You Z., Liu Q., Li C., Tang R., Wu S. (2025). Dynamic synthesis and transport of phenazine-1-carboxylic acid to boost extracellular electron transfer rate. Nat. Commun..

[13] Cao B., Zhao Z., Peng L., Shiu H.Y., Ding M., Song F., Guan X., Lee C.K., Huang J., Zhu D. (2021). Silver nanoparticles boost charge-extraction efficiency in *Shewanella* microbial fuel cells. Science.

[14] Chen H., Simoska O., Lim K., Grattieri M., Yuan M., Dong F., Lee Y.S., Beaver K., Weliwatte S., Gaffney E.M. (2020). Fundamentals, applications, and future directions of bioelectrocatalysis. Chem. Rev..

[15] Fan X., Zhou Y., Jin X., Song R.B., Li Z., Zhang Q. (2021). Carbon material-based anodes in the microbial fuel cells. Carbon Energy.

[16] Jiang B., Jiang N., Cui Y., Wang H., Zhang G., Li J., Zhang Y. (2024). Rapid Synthesis and Microenvironment Optimization of Hierarchical Porous Fe-N-C Catalysts for Enhanced ORR in Microbial Fuel Cells. Adv. Sci..

[17] Yang C.-L., Wang L.-N., Yin P., Liu J., Chen M.-X., Yan Q.-Q., Wang Z.-S., Xu S.-L., Chu S.-Q., Cui C. (2021). Sulfur-anchoring synthesis of platinum intermetallic nanoparticle catalysts for fuel cells. Science.

[18] Xu Z., Tan X., Chen C., Wang X., Sui R., Zhuang Z., Zhang C., Chen C. (2024). Recent advances in microenvironment regulation for electrocatalysis. Natl. Sci. Rev..

[19] Santoro C., Bollella P., Erable B., Atanassov P., Pant D. (2022). Oxygen reduction reaction electrocatalysis in neutral media for bioelectrochemical systems. Nat. Catal..

[20] Zhou X., Wu D., Zhang Y., Feng T., Zhang W., Zhang Z. (2025). Inorganic-bacterial biohybrids for efficient solar-driven nitrogen fixation. Nat. Commun..

[21] Su L., Fukushima T., Prior A., Baruch M., Zajdel T.J., Ajo-Franklin C.M. (2020). Modifying Cytochrome c Maturation Can Increase the Bioelectronic Performance of Engineered *Escherichia coli*. ACS Synth. Biol..

[22] Niu Y., Guo G., Xue D., Yang X., Dai X., Bai Z., Yang L. (2024). Modifying cellular properties via rational chemical design for unnatural oxygen reduction electrocatalysis of a cell. J. Mater. Chem. A.

[23] Xu B., Chen B.-Y., Hsueh C.-C., Qin L.-J., Chang C.-T. (2014). Deciphering characteristics of bicyclic aromatics-mediators for reductive decolorization and bioelectricity generation. Bioresour. Technol..

[24] Chen B.-Y., Xu B., Yueh P.-L., Han K., Qin L.-J., Hsueh C.-C. (2015). Deciphering electron-shuttling characteristics of thionine-based textile dyes in microbial fuel cells. J. Taiwan Inst. Chem. Eng..

[25] Chen B.-Y., Liao J.-H., Hsu A.-W., Tsai P.-W., Hsueh C.-C. (2018). Exploring optimal supplement strategy of medicinal herbs and tea extracts for bioelectricity generation in microbial fuel cells. Bioresour. Technol..

[26] Niu Y., Xue D., Dai X., Guo G., Yang X., Yang L., Bai Z. (2024). Sustainable power generation from sewage with engineered microorganisms as electrocatalysts. Nat. Sustain..

[27] Madondo N.I., Rathilal S., Bakare B.F., Tetteh E.K. (2023). Effect of electrode spacing on the performance of a membrane-less microbial fuel cell with magnetite as an additive. Molecules.

[28] Guo M., Lu X., Qiao S. (2024). Nitrate removal by anammox bacteria utilizing photoexcited electrons via inward extracellular electron transfer channel. Water Res..

[29] Wang Q., Kim H., Halvorsen T.M., Chen S., Hayes C.S., Buie C.R. (2023). Leveraging microfluidic dielectrophoresis to distinguish compositional variations of lipopolysaccharide in *E. coli*. Front. Bioeng. Biotechnol..

[30] Díaz Calvo T., Tejera N., McNamara I., Langridge G.C., Wain J., Poolman M., Singh D. (2022). Genome-scale metabolic modelling approach to understand the metabolism of the opportunistic human pathogen *Staphylococcus epidermidis* RP62A. Metabolites.

[31] Antunes J.M., Silva M.A., Salgueiro C.A., Morgado L. (2022). Electron flow from the inner membrane towards the cell exterior in *Geobacter sulfurreducens*: Biochemical characterization of cytochrome CbcL. Front. Microbiol..

[32] Xu B., Lan J.C., Sun Q., Hsueh C., Chen B.-Y. (2019). Deciphering optimal biostimulation strategy of supplementing anthocyanin-abundant plant extracts for bioelectricity extraction in microbial fuel cells. Biotechnol. Biofuels.

[33] Sun J., Jin S., Wang Z. (2025). MitoQ alleviates mitochondria damage in sepsis-acute lung injury in a citrate synthase dependent manner. Inflamm. Res..

[34] Chen H., Bai Z., Dai X., Zeng X., Cano Z.P., Xie X., Zhao M., Li M., Wang H., Chen Z. (2019). In situ engineering of intracellular hemoglobin for implantable high-performance biofuel cells. Angew. Chem..

[35] Fan S., Liang B., Xiao X., Bai L., Tang X., Lojou E., Cosnier S., Liu A. (2020). Controllable display of sequential enzymes on yeast surface with enhanced biocatalytic activity toward efficient enzymatic biofuel cells. J. Am. Chem. Soc..

[36] Lin C.-W., Lai C.-Y., Liu S.-H., Chen Y.-R., Alfanti L.K. (2021). Enhancing bioelectricity generation and removal of copper in microbial fuel cells with a laccase-catalyzed biocathode. J. Clean. Prod..

[37] Li H., Zhang X., Qin Y., Liu Y., Wang J., Peng L., Li C. (2021). Crafting controllable Fe-based hierarchically organic-frameworks from bacterial cellulose nanofibers for efficient electrocatalysts in microbial fuel cells. J. Power Sources.

[38] Han H.H., Jung S.-M., Kim S.-K., Lee G.-H., Kim S.-J., Kim Y.-T., Hahn S.K. (2022). Bimetallic electrocatalyst of hyaluronate-Au@ Pt for durable oxygen reduction in biofuel cells. ACS Appl. Energy Mater..

[39] Zhao T., Fang J., Niu Y., Zhu K., Wang L., Pan J., Liu C., Shi W., Li Y., Wang X. (2026). Trifunctional endogenous mediator orchestrates efficient biocathodes via synergistic electron transfer and enzyme catalytic site modulation. Nano Res..

[40] Chaudhuri S.K., Lovley D.R. (2003). Electricity generation by direct oxidation of glucose in mediatorless microbial fuel cells. Nat. Biotechnol..

[41] Choi D., Lee S.B., Kim S., Min B., Choi I.-G., Chang I.S. (2014). Metabolically engineered glucose-utilizing *Shewanella* strains under anaerobic conditions. Bioresour. Technol..

[42] Lin T., Bai X., Hu Y., Li B., Yuan Y.J., Song H., Yang Y., Wang J. (2017). Synthetic *Saccharomyces cerevisiae-Shewanella oneidensis* consortium enables glucose-fed high-performance microbial fuel cell. AIChE J..

[43] Bai X., Lin T., Liang N., Li B.-Z., Song H., Yuan Y.-J. (2021). Engineering synthetic microbial consortium for efficient conversion of lactate from glucose and xylose to generate electricity. Biochem. Eng. J..

[44] Sivakumar K., Scarascia G., Zaouri N., Wang T., Kaksonen A.H., Hong P.-Y. (2019). Salinity-Mediated Increment in Sulfate Reduction, Biofilm Formation, and Quorum Sensing: A Potential Connection Between Quorum Sensing and Sulfate Reduction? *Front*. Microbiol..

[45] Lee S.Y., Choi J.W., Lee T.G., Heo M.B., Son J.G. (2024). Influence of albumin concentration on surface characteristics and cellular responses in the pre-incubation of multi-walled carbon nanotubes. Nanoscale Adv..

[46] Ma F., Gao T., Sun X., Han C., Wang Y., Jiang A., Zhou Y., Liang G., Wang H., Wang L. (2025). Keto-enol tautomerism as dynamic electron/hole traps promote charge carrier separation for hydrogen peroxide photosynthesis. Nat. Commun..

[47] Mathialagan S.K., Parreiras S.O., Tenorio M., Černa L., Moreno D., Muñiz-Cano B., Navío C., Valvidares M., Valbuena M.A., Urgel J.I. (2024). On-Surface Synthesis of Organolanthanide Sandwich Complexes. Adv. Sci..

[48] Wu W., Cui Y., Wu Y., Ni Y., Zhao C., Sun W., Yi Q. (2025). Epigenetic roles of chromatin remodeling complexes in bone biology and the pathogenesis of bone-related disease. Int. J. Mol. Med..

[49] Pandit C., Thapa B.S., Srivastava B., Mathuriya A.S., Toor U.-A., Pant M., Pandit S., Jadhav D.-A. (2022). Integrating human waste with microbial fuel cells to elevate the production of bioelectricity. BioTech.

