# Supplementary material for: Anthocyanin Boosts Electroactive Biofilms Formation and Regulates Intrinsic Catalytic Activity of Single Cells in Escherichia coli for Sustainable Bio-Electrocatalysis in Microbial Fuel Cells
Source: Microorganisms. 2026 Apr 13;14(4):872. doi: 10.3390/microorganisms14040872 (PMC13119018; doi:10.3390/microorganisms14040872)
Supplement: Supplementary file 1 [file microorganisms-14-00872-s001.zip › microorganisms-4222850-supplementary.pdf]

microorganisms

MDPI

Article

Anthocyanin Boosts Electroactive Biofilms Formation and Regulates Intrinsic Catalytic Activity of Single Cells in *Escherichia coli* for Sustainable Bio-Electrocatalysis in Microbial Fuel Cells

Kai Zong [1,2,*], Liwen Chen [1,2], Waseem Raza [1,2], Xin Wang [1,2], Lin Yang [1,2] and Zhongwei Chen [3,4,*]

[1] Institute of Carbon Neutrality, Zhejiang Wanli University, Ningbo 315100, China; chenliwen0903@163.com (L.C.); razawaseem2@zwu.edu.cn (W.R.); wangx@zwu.edu.cn (X.W.); yanglin@htu.edu.cn (L.Y.)
[2] Ningbo Key Laboratory of High Energy Density Battery, Yuyao Innovation Institute, Zhejiang Wanli University, Ningbo 315400, China
[3] Power Battery and System Research Center, Dalian Institute of Chemical Physics, Chinese Academy of Sciences, Dalian 116023, China
[4] State Key Laboratory of Catalysis, Dalian Institute of Chemical Physics, Chinese Academy of Sciences, Dalian 116023, China
* Correspondence: zongkai@zwu.edu.cn (K.Z.); zwchen@dicp.ac.cn (Z.C.)

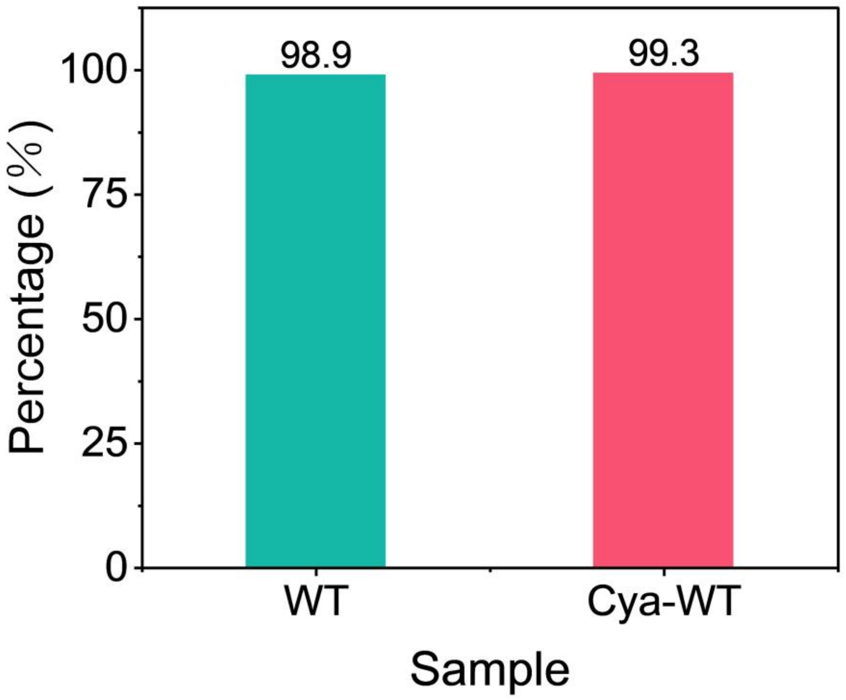

Figure S1. The survival rate graph was obtained based on Imaris processing of WT and Cya-WT.

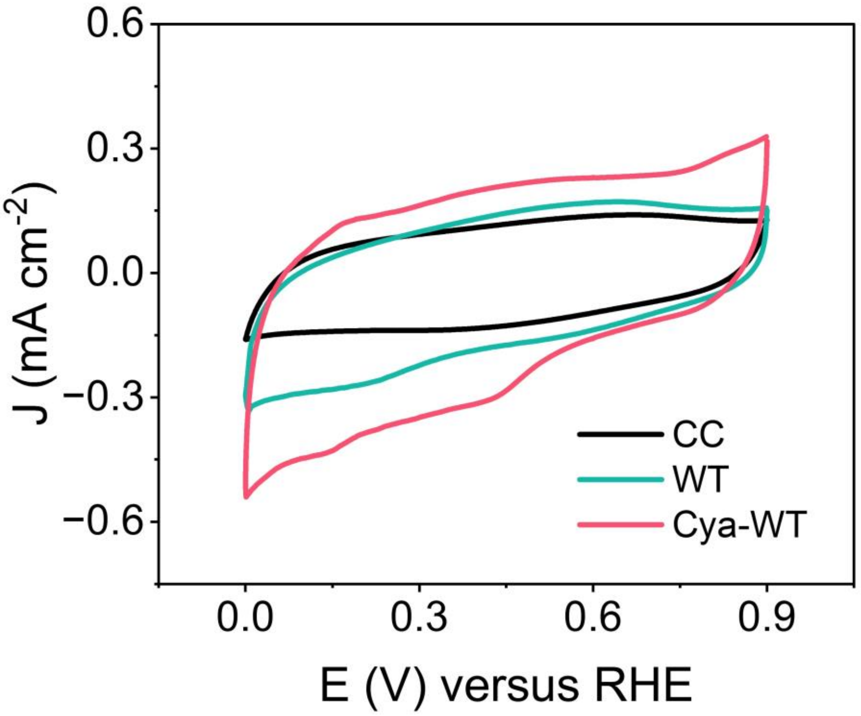

Figure S2. CV patterns of the CC electrode, WT cell, and Cya-WT cell in N_2-saturated solution.

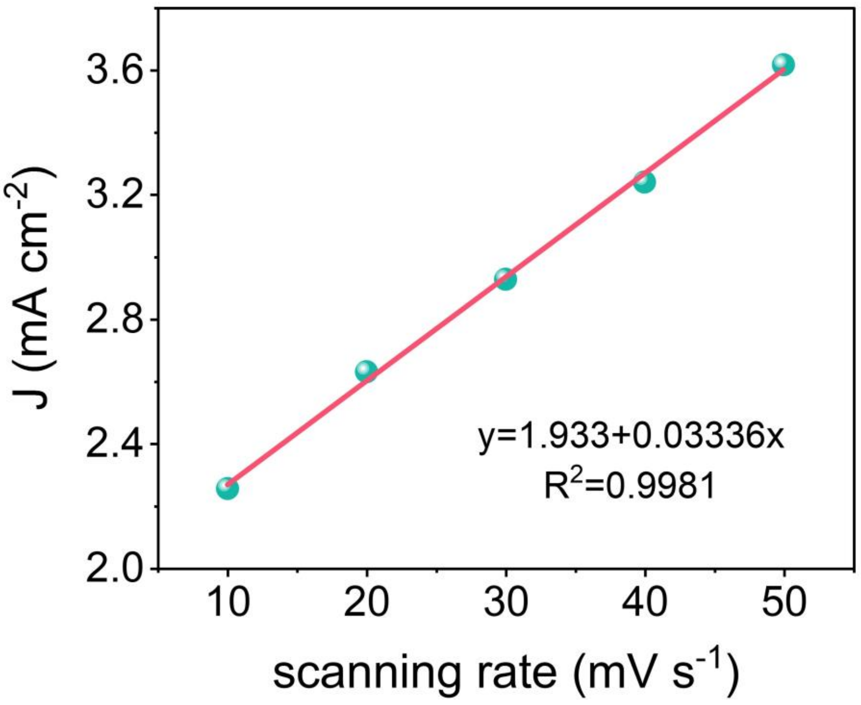

Figure S3. Linear relationship plot between cathode peak current and scanning rate.

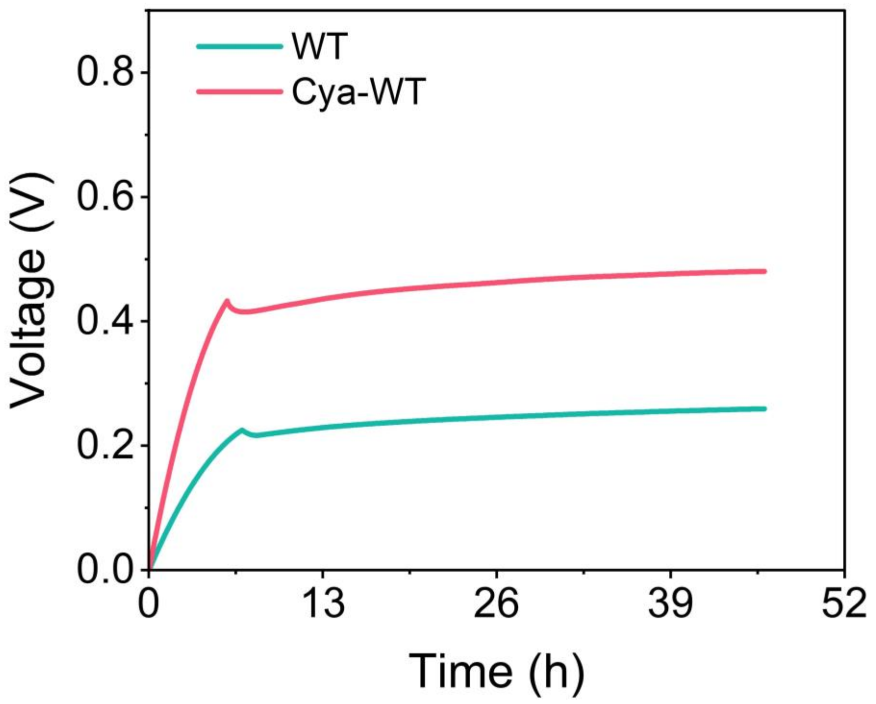

Figure S4. Startup voltage diagram of the MFC assembled using the CC biocathodes loaded with different *E. coli* loads.

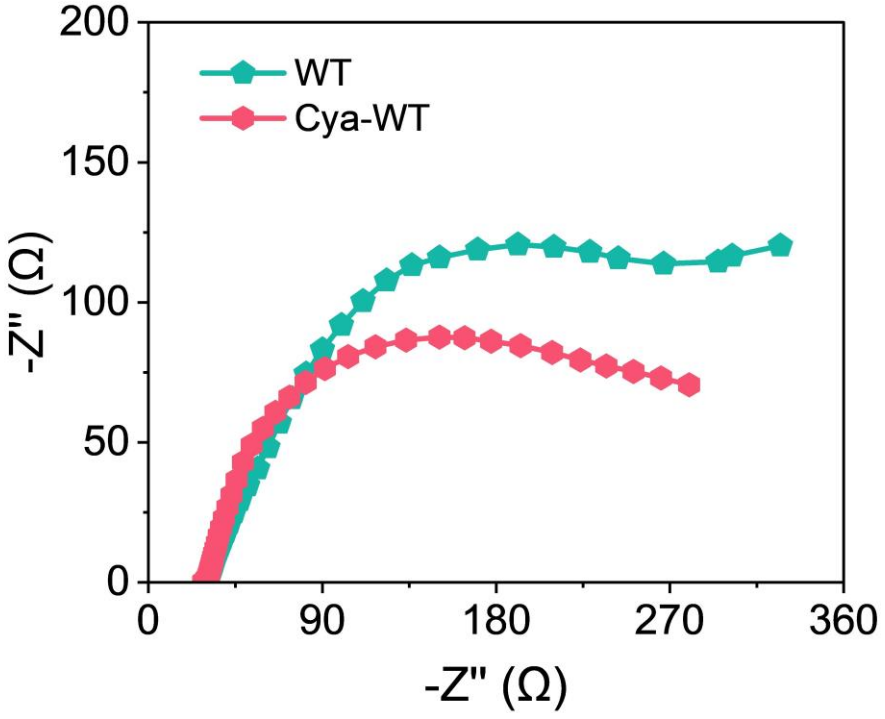

Figure S5. EIS diagram of the MFC assembled using the CC biocathodes loaded with different *E. coli* loads.
